# Identification of a common secondary mutation in the *Neurospora crassa* knockout collection conferring a cell fusion-defective phenotype

**DOI:** 10.1128/spectrum.02087-23

**Published:** 2023-08-25

**Authors:** Alejandro Montenegro-Montero, Alejandra Goity, Paulo F. Canessa, Luis F. Larrondo

**Affiliations:** 1 Departamento de Genética Molecular y Microbiología, Facultad de Ciencias Biológicas, Pontificia Universidad Católica de Chile, Santiago, Chile; 2 Agencia Nacional de Investigación y Desarrollo-Millennium Science Initiative Program, Millennium Institute for Integrative Biology, Santiago, Chile; 3 Centro de Biotecnología Vegetal, Facultad de Ciencias de la Vida, Universidad Andrés Bello, Santiago, Chile; University of Michigan-Michigan Medicine, Ann Arbor, Michigan, USA

**Keywords:** *Neurospora crassa*, cell fusion, gene knockout, secondary mutations

## Abstract

Gene-deletion mutants represent a powerful tool to study gene function. The filamentous fungus *Neurospora crassa* is a well-established model organism, and features a comprehensive gene knockout strain collection. While these mutant strains have been used in numerous studies, resulting in the functional annotation of many Neurospora genes, direct confirmation of gene-phenotype relationships is often lacking, which is particularly relevant given the possibility of background mutations, sample contamination, and/or strain mislabeling. Indeed, spontaneous mutations resulting in phenotypes resembling many cell fusion mutants have long been known to occur at relatively high frequency in *N. crassa*, and these secondary mutations are common in the Neurospora deletion collection. The identity of these mutations, however, is largely unknown. Here, we report that the Δ*ada-3* strain from the *N. crassa* knockout collection, which exhibits a cell fusion defect, harbors a secondary mutation responsible for this phenotype. Through whole-genome sequencing and genetic analyses, we found a ~30-Kb deletion in this strain affecting a known cell fusion-related gene, *so*/*ham-1*, and show that it is the absence of this gene—and not of *ada-3*—that underlies its cell fusion defect. We additionally found three other knockout strains harboring the same deletion, suggesting that this mutation may be common in the collection and could have impacted previous studies. Our findings provide a cautionary note and highlight the importance of proper functional validation of strains from mutant collections. We discuss our results in the context of the spread of cell fusion-defective cheater variants in *N. crassa* cultures.

**IMPORTANCE:**

This study emphasizes the need for careful and detailed characterization of strains from mutant collections. Specifically, we found a common deletion in various strains from the *Neurospora crassa* gene knockout collection that results in a cell fusion-defective phenotype. This is noteworthy because this collection is known to contain background mutations—of a largely unclear nature—that produce cell fusion-defective phenotypes. Our results describe an example of such mutations, and highlight how this common genetic defect could have impacted previous studies that have used the affected strains. Furthermore, they provide a cautionary note about the use of Neurospora strains with similar phenotypes. Lastly, these findings offer additional details relevant to our understanding of the origin and spread of cell fusion-defective cheater variants in *N. crassa* cultures.

## INTRODUCTION

The orange bread mold *Neurospora crassa* has a vast and rich history as a model system (1, 2), and is perhaps the best understood filamentous fungus. Because of ease of culture, facile genetics, rapid growth rate, and an available genome sequence [encoding about 10,000 protein-coding genes (3)], together with an expanding molecular toolkit (4 – 6), Neurospora has contributed to key findings in several research fields and continues to represent a powerful model, particularly for animal and plant pathogens, and for agriculturally and industrially relevant species (7, 8). As such, and building on decades of research, a functional genomics characterization of *N. crassa* has the potential to greatly contribute to our understanding of eukaryotic biology.

To maximize this potential, a consortium of laboratories working on Neurospora set out to systematically delete, via targeted gene replacement, all of the predicted protein-coding genes in this organism (9). Starting with the initial report in 2006 (10), this Neurospora Functional Genomics Project created full deletion mutants for ~9,000 of its predicted protein-coding genes [all of which are readily available from the Fungal Genetics Stock Center (11)], creating an invaluable resource to characterize gene function in this model system. Part of this project also involved phenotypic analysis of the resulting mutants, and large-scale screening studies have generated phenotypic data for nearly 1,300 *N*. *crassa* knockout (KO) strains (12 – 16), with the corresponding functional annotations.

Generation of a mutant strain followed by evaluation of the resulting phenotype is a standard and powerful approach to study gene function. A key consideration when pursuing such a strategy, however, is the existence of secondary mutations in the strain of interest that can confound the results. Indeed, it has been reported in multiple systems that laboratory strains and mutant collections exhibit considerable nucleotide variation and secondary (i.e., background) mutations (17 – 22), which can impact the conclusions drawn from studies that use them. In *N. crassa*, it has long been observed that spontaneous mutations resulting in a phenotype characterized by altered asexual development and female sterility, reminiscent of that exhibited by many cell fusion-defective mutants (23), arise frequently (24). Indeed, the Free group has reported the existence of hundreds of strains in the Neurospora KO collection that exhibit a similar pleiotropic phenotype that appears to be unrelated to the absence of the target gene (25, 26). The nature of the mutation(s) underlying this phenotype, however, is largely unknown.

A common approach to evaluating whether an observed phenotype is due to the absence of a particular gene in a KO strain in Neurospora is to perform co-segregation assays. Here, the KO strain of interest is typically back-crossed to a parental wild-type (WT) strain, and the progeny is then scored to evaluate whether the knockout cassette (i.e., the cassette used for gene replacement, harboring the selection marker) co-segregates with the phenotype. A limitation of this assay, however, is that if a secondary mutation is responsible for the phenotype, and it is located in close proximity to the target gene locus, it may be difficult to find the recombinant strains, unless a large number of progeny is scored. A second approach to confirm whether a particular gene is responsible for an observed phenotype, is simply re-introducing a WT copy of the deleted gene and evaluating whether the phenotype is reversed (i.e., complementation assay). While several phenotypic studies have been done using the Neurospora KO collection, many of the reported phenotypes remain unvalidated, that is, no segregation and/or complementation assays have been reported to confirm that the target gene in the KO strain is indeed responsible for the observed phenotype.

In this study, we report that the Δ*ada-3* strain from the *N. crass*a KO collection, which exhibits a pleiotropic phenotype commonly associated with cell fusion-defective mutants, harbors a secondary mutation responsible for this phenotype. Indeed, through whole-genome sequencing and genetic analyses, we found a ~30-Kb deletion that affects a known cell fusion-related gene, *so/ham-1*, and show that it is deletion of this gene—and not of *ada-3*—that is responsible for the cell fusion-defective phenotype in this strain. We additionally found three other knockout strains that harbor the same deletion, suggesting that this mutation may be common in the collection. Our results highlight the importance of proper functional validation of strains from the *N. crassa* KO collection and suggest that conclusions of studies based on the strains herein reported—or on others with similar phenotypes—may need to be reevaluated. In addition, our findings complement recent studies on fusion-defective cheater lineages and their relationship to the long-standing observation of the frequent emergence of *soft*-like morphological mutants in *N. crassa* laboratory cultures.

## MATERIALS AND METHODS

### *N. crassa* strains and culturing

General conditions for *N. crassa* growth and maintenance, as well as routine manipulative procedures, followed those described by Davis and De Serres (27). Strains were maintained at 25°C on Vogel’s minimal medium (28) with 2% wt/vol sucrose and 2% wt/vol agar (hereafter referred to as VMM). Growth tube assays to determine growth rate (29) were done on the same medium. Crosses were performed on Westergaard and Mitchell’s synthetic crossing medium (30). For ascospore germination and subsequent isolation, sorbose-containing medium (FIGS [fructose-inositol-glucose-sorbose]) was used (27). Picked ascospores were then grown at 25°C on VMM, supplemented with hygromycin (200 µg/mL; Calbiochem, San Diego, CA, USA) when necessary.

**Fig 1:**
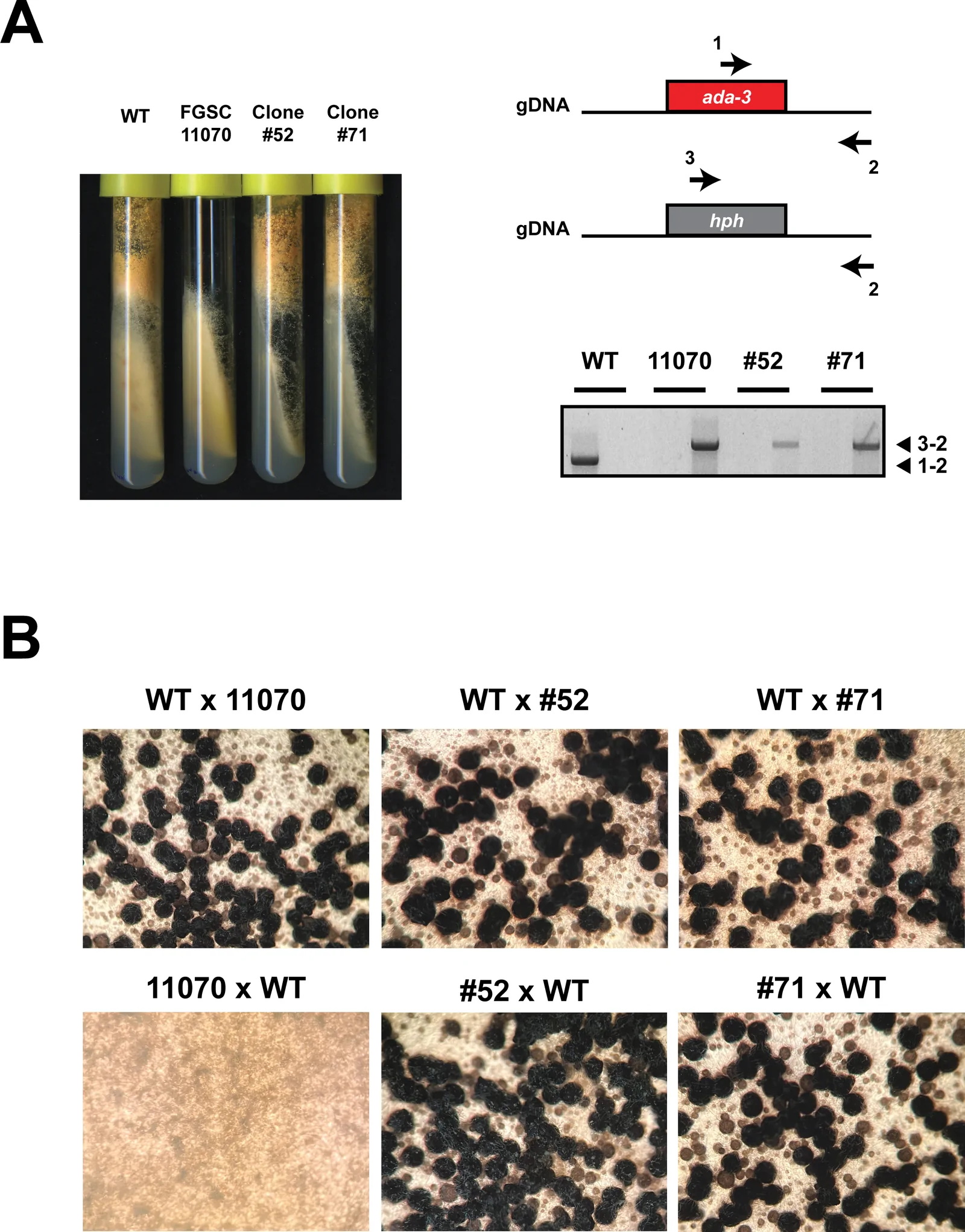
The absence of *ada-3* is not responsible for the ADA phenotype of FGSC 11070. (**A**) (left) Strains were imaged after culture on slants of VMM for 7 days at 25°C under constant light. Clones #52 and #71 (Δ*ada-3*, a) were obtained by crossing 11070 with a WT strain (a *his-3*+ derivative of FGSC 9720, as female), after which germinated ascospores were individually transferred to slants of VMM + 200 µg/mL hygromycin, as described in Materials and Methods. (Right) Genomic DNA (gDNA) was extracted from the strains listed, and PCR was used to evaluate the presence of either *ada-3* or the *hph* cassette at the *ada-3* locus. Note that even though both reactions were tested per homokaryotic strain, each strain can only be positive for either the WT gene or the KO cassette, but not both. The arrows denote the position of the primers used (see Table 1). (**B**) For crosses, strains used as females were first grown on synthetic crossing medium for 7 days at 25°C under constant light, after which they were fertilized with conidia from different strains with the opposite mating type (i.e., males) and incubated under the same conditions for an additional 7 days before imaging. Reciprocal crosses were set up for each strain pair, so that every strain could be tested for both female and male fertility. For every cross, the strain used as female is listed first (i.e., female x male).

Unless otherwise noted, WT and KO *N. crassa* strains were obtained from the Fungal Genetics Stock Center (http://www.fgsc.net/). Unless otherwise specified, strains FGSC 2489 (74-OR23-1VA) and FGSC 988 (74-OR8-1a) were used as WT for all experiments. The KO strains used were generated as part of the Neurospora Functional Genomics Project (9, 10), in which each target open reading frame is replaced with a hygromycin B phosphotransferase gene (*hph*) cassette, which confers resistance to hygromycin. All KO strains used in this study were verified by PCR, by checking that the target gene is indeed absent and replaced by the knockout cassette. For co-segregation experiments with FGSC 11070 (Δ*NCU02896*, a), a standard *his-3*+ derivative of FGSC 9720 was used as WT for mating (see Fig. 1A) (10, 31).

Images of phenotypic assays are representative of more than five independent experiments, unless otherwise noted.

### Genomic DNA isolation

For genomic DNA isolation, conidia were inoculated in 4 mL of liquid Vogel’s minimal medium with 2% wt/vol glucose and grown at 30°C for 24 h in constant light, with constant shaking (200 rpm). Mycelia were harvested, dried, and snap-frozen, and then ground in liquid nitrogen with a mortar and pestle. Genomic DNA was then extracted using the Puregene DNA Isolation Kit (D-7000A, Gentra Systems) according to the manufacturer’s instructions.

### Sequencing and analysis

For whole-genome sequencing, Illumina libraries were prepared using the Nextera DNA Library Prep Kit (Illumina), and the manufacturer’s protocol was followed with one exception—we performed agarose size selection of the Nextera libraries, extracting the ~500 bp bands. Multiplexed libraries were then sequenced on an Illumina HiSeq 2,500 system, with 100-bp paired-end reads. Sequencing data (FASTQ files) were first demultiplexed, and quality was then inspected using FastQC (https://www.bioinformatics.babraham.ac.uk/projects/fastqc/). Reads were trimmed using BBDuk (version 38.18, https://sourceforge.net/projects/bbmap/) to remove adapter sequences and low-quality reads. Only paired read mates with a quality Q score ≥30 were further processed. Sequences were then mapped to the *N. crassa* OR74A NC12 reference genome (assembly GCA_000182925.2) using the short-read aligner BWA-MEM (version 0.7.17-r1188), with default parameters (32). BAM alignment files were then sorted and indexed with SAMtools (33), version 1.14. Lastly, sorted files were visually inspected using the Integrative Genome Viewer (IGV) (34), version 2.11.9.

For sequencing the *so*/*ham-1* locus in strains of interest, we amplified the relevant region from genomic DNA using a nested PCR approach (Table 1), and performed full-length amplicon sequencing using the Oxford Nanopore Technologies platform, as provided by Plasmidsaurus (Eugene, OR, USA)

**TABLE 1:** Primers used in this study

Primer name	Description	Sequence (5´ to 3´)
1	Within *ada-3* (for genotyping)	CCAGGGCCAAACATGAATAC
2	Downstream *ada-3* (for genotyping)	GTCTAAGGCGCCACGTTAAG
3	Within *hph* (forward, for genotyping)	GCCATGTAGTGTATTGACCG
oL2072	Upstream the ~30-Kb deletion. For deletion validation via Sanger sequencing	GATGAAGGGGATGGACAAGA
oL2073	Downstream the ~30-Kb deletion. For deletion validation via Sanger sequencing	AGAATCGAGGGAGAAGAGCA
oL2074	Within *so/ham-1* (for genotyping)	AACCTACGGCAACCCTCTTG
oL2075	Within *so/ham-1* (for genotyping)	TTGGTGTCGGAGACTTCGTG
oL3433	Upstream the ~30-Kb deletion. For genotyping (forward, round 1 of nested PCR)	ATCCAGACCGGACTGACTGA
oL3434	Downstream the ~30-Kb deletion. For genotyping (reverse, round 1 of nested PCR)	ATCCGATTGGGTACATGGGG
oL6804	Upstream the ~30-Kb deletion. For genotyping (forward, round 2 of nested PCR	TTGGCGCTGTAGTCGGATAC
oL6806	Downstream the ~30-Kb deletion. For genotyping (reverse, round 2 of nested PCR)	TCCCGCTCAATTGGCTTGAT
4	Within *acw-4* (for genotyping)	CAGAAGAAACCCATTAAGTC
5	Downstream *acw-4* (for genotyping)	TCACTCGCACCAACGTCGTG
6	Upstream *rco-1* (for genotyping)	ATGAAGCAAAACCGAACCTG
7	Within *rco-1* (for genotyping)	CTATAGACGCTGTCCTTGTGG
8	Within *hph* (reverse, for genotyping)	ACTGTCGGGCGTACACAAAT
oL6873	For sequencing the *so*/*ham-1* locus (forward, round 1 of nested PCR)	AGTCGGCTGCTTGAAAGGTA
oL6863	For sequencing the *so*/*ham-1* locus (reverse, round 1 of nested PCR)	AGAGAGACGCTTTGCTTGCT
oL6862	For sequencing the *so*/*ham-1* locus (forward, round 2 of nested PCR)	TGCAACTGCTCTCGTCCAAT
oL6864	For sequencing the *so*/*ham-1* locus (reverse, round 2 of nested PCR)	GAACACACATTGCCGACATC

### Complementation assay

Complementation experiments were done with a *his-3*-targeting vector system, using plasmids that allow for the expression of *so/ham-1* under the control of either the *ccg-1* promoter or its native promoter (pCCG1-SO-GFP or pSO-SO-GFP, respectively, courtesy of N. Louise Glass). These plasmids were created using pMF272 as a backbone, which is derived from pBM60/pBM61 (35). As such, the empty *his-3*-targeting vector pBM61 was used as a control. For complementation, strain FGSC 11070 (Δ*NCU02896*, a) was crossed to FGSC 9720 (Δ*mus-52::bar+, his-3,* A), and *his-3* progeny displaying the altered asexual development phenotype characteristic of FGSC 11070 [“flat” conidiation pattern and short aerial hyphae (10)] was then used for transformation with the aforementioned plasmids. Transformants were then picked and grown on VMM.

## RESULTS

### The absence of *ada-3* is not responsible for the ADA phenotype of FGSC 11070

One of the strains generated by the Neurospora knockout project is FGSC 11070. This strain was first reported and characterized by Colot et al. (10), and was shown to exhibit an “all development altered” (ADA) phenotype, as it displayed defects in all the traits therein examined, namely basal hyphal extension, asexual development, and sexual development. The knocked out gene, which encodes for a putative Zn cluster transcription factor (36), was termed *ada-3* (*NCU02896*). The FGSC 11070 strain exhibits a pleiotropic phenotype, featuring a “flat” conidiation pattern, short aerial hyphae, and female sterility (10, 13), resembling a phenotype typically displayed by cell fusion-defective strains (23). Indeed, it has been shown that FGSC 11070 additionally fails to produce conidial anastomosis tubes (CATs), and ADA-3 has thus been suggested to play a role in cell fusion (25).

Upon genetic studies using the FGSC 11070 (Δ*ada-3*) strain (31), we noticed that the abnormal asexual development phenotype of this strain did not co-segregate with the hygromycin-resistance cassette and, hence, with the lack of *ada-3*. Indeed, when crossing 11070 with a WT strain (using WT as female, as 11070 is female sterile) and then scoring >55 single ascospore isolates, we were able to occasionally find progeny that, while it lacked the *ada-3* gene (and was hygromycin resistant, HygR), it displayed a WT phenotype (Fig. 1A). This suggested that the phenotype displayed by 11070 is not a consequence of the absence of *ada-3*. Consistent with this, the WT-like Δ*ada-3* progeny (i.e., the Δ*ada-3* progeny that displays a WT phenotype) is also fully fertile, as opposed to 11070, which is female sterile (Fig. 1B). Together, these results suggest that the reported ADA phenotype exhibited by 11070 is not due to the absence of *ada-3* but is, instead, a consequence of a secondary mutation.

### The ADA phenotype of FGSC 11070 is due to the absence of *so/ham-1*

To identify the genetic alteration responsible for the pleiotropic phenotype displayed by 11070, we performed whole-genome sequencing. To do this, we analyzed the progeny from a cross between 11070 and a WT strain (Fig. 1A). Given that the hygromycin-resistance cassette does not co-segregate with the phenotype (and, thus, the mutation of interest), we selected Δ*ada-3* (i.e., HygR) progeny that displayed either a WT or an ADA phenotype, for comparison. We then picked four clones per group, extracted DNA, and subjected each sample individually to high-throughput Illumina DNA sequencing.

**Fig 2:**
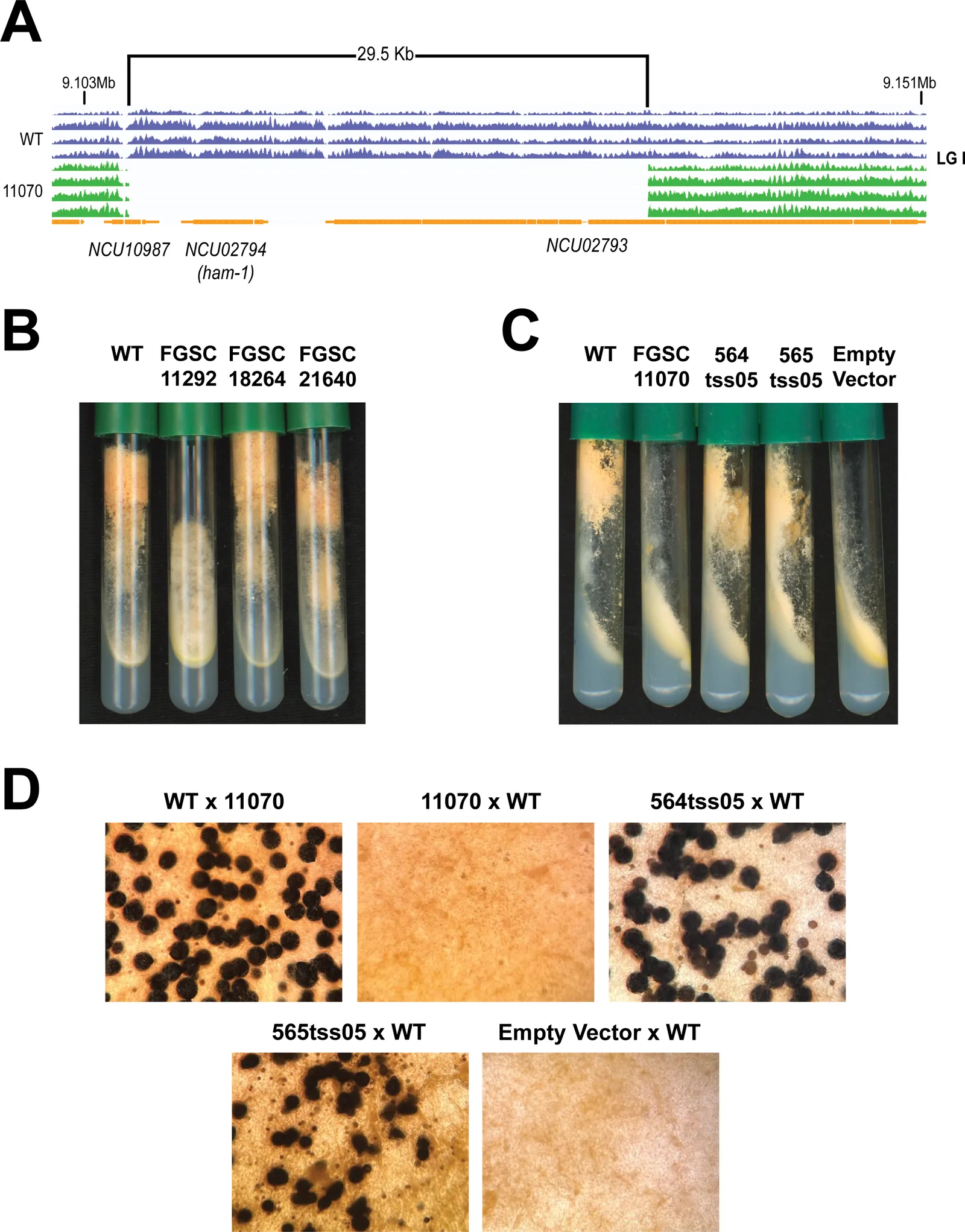
The ADA phenotype of FGSC 11070 is due to the absence of *so/ham-1*. (**A**) Integrative Genomics Viewer image of a portion of linkage group I (LG I), showing coverage tracks (blue, green) for the eight *N. crassa* strains sequenced and the corresponding location of annotated genes (orange). Four independent WT-like Δ*ada-3* strains (blue, WT) and four independent ADA-like Δ*ada-3* strains (green, 11070) were sequenced. (**B**) Phenotypic analysis of candidate strains. The strains shown were imaged after culture on slants of VMM for 7 days at 25°C under constant light. FGSC 11292, 18264, and 21640 correspond to full deletion strains of *NCU02794*, *NCU02793*, and *NCU10987*, respectively. (**C**) Complementation assay. WT, 11070, and a 11070 *his-3* strain transformed with Pccg-1-so-GFP (564tss05), Pso-so-GFP (565tss05), or pBM61 (empty vector) (see Materials and Methods), were grown on slants of VMM for 7 days at 25°C under constant light before imaging. (**D**) The same strains as in (**C**) were tested for female fertility. To do that, the strains were first grown on synthetic crossing medium for 7 days at 25°C under constant light, after which they were fertilized with conidia from WT of the opposite mating type (i.e., males). The crosses were incubated under the same conditions for an additional 7 days before imaging. For every cross, the strain used as female is listed first (i.e., female × male).

The approximately 40 Mb *N*. *crassa* genome is organized in seven chromosomes (linkage groups LG I–LG VII). Analysis of the sequencing data showed that the clones with the ADA phenotype all have a ~30-Kb (29,585 bp) deletion in LG I that physically affects three annotated genes, namely *NCU10987, NCU02794*, and *NCU02793* (Fig. 2A). We confirmed the presence and identity of this deletion via PCR with primers flanking the predicted missing region and by cloning followed by Sanger sequencing, respectively (Table 1). In addition, and as expected, the whole-genome sequencing data confirmed the Δ*ada-3* status of all sequenced clones.

To evaluate whether any of the genes affected by the deletion is responsible for the ADA phenotype in 11070, we characterized individual deletion strains of each of the three genes, to see if any of these single-gene mutant strains exhibits the ADA phenotype. We obtained the corresponding KO strains from the FGSC and then monitored their growth under standard conditions. We observed that while the FGSC 21640 (Δ*NCU10987*) and FGSC 18264 (Δ*NCU02793*) strains exhibit a WT phenotype, deletion of *NCU02794* (FGSC 11292) results in the ADA phenotype (Fig. 2B), which suggests that the absence of this gene is responsible for the mutant phenotype in 11070.

The disrupted gene in 11292 is *so/ham-1*, which has been reported to play a role in cell fusion in Neurospora (37, 38). Similar to many cell fusion mutants, the Δ*so/ham-1* strain exhibits an abnormal development, characterized by a flat, carpet-like phenotype with shortened aerial hyphae, an altered conidiation pattern, and female sterility, which is consistent with the phenotype displayed by 11070, further supporting the idea that the phenotype in 11070 is a result of the absence of *so/ham-1*. To directly evaluate this, we introduced a WT copy of *so/ham-1* into the *his-3* locus of a 11070 *his-3* strain harboring the ~30-Kb deletion (see Materials and Methods). We observed that expression of *so/ham-1* was able to rescue both the flat conidiation phenotype (Fig. 2C) and the female sterility (Fig. 2D) exhibited by the ~30-Kb deletion mutant, suggesting that the phenotype in this strain is indeed due to the lack of *so/ham-1*.

Together, these results confirm that 11070 harbors a secondary mutation (independent from the *ada-3* deletion), that this secondary alteration corresponds to a ~30-Kb deletion in LG I, that this deletion affects multiple genes —including *so/ham-1—*, and that the flat conidiation phenotype of 11070 is not a consequence of the absence of *ada-3* but of the lack of *so/ham-1*. In addition, these results suggest that ADA-3 is not involved in cell fusion in *N. crassa* as previously suggested, and that studies that have proposed a role of this putative transcription factor in cell fusion are confounded due to using a Δ*ada-3* strain that additionally harbors a deletion in a known cell fusion protein, SO/HAM-1, whose deletion—on its own—results in cell fusion defects (37, 38) (see Discussion).

### The ~30-Kb deletion is present in multiple strains of the knockout collection

The identification of a secondary mutation in a strain of the *N. crassa* KO collection that is responsible for a flat conidiation phenotype was considered particularly relevant, given both that many deletion strains in the collection exhibit such phenotype (10, 13, 15, 16)—in many cases due to secondary mutations (25)—and that no segregation analysis or complementation studies have been done for most of these deposited mutant strains. This suggested to us that the secondary mutation we identified might be present in multiple strains in the collection and, importantly, be responsible for their reported phenotype.

A study by Fu et al. set out to identify genes required for cell-to-cell fusion in *N. crassa* (25). The authors screened the KO collection for putative cell fusion mutants by initially looking at strains that exhibited a flat conidiation pattern and a defect or delay in the production of protoperithecia, a phenotype common to many cell fusion mutants (23). This was followed by subsequent segregation, complementation, and/or functional assays, which, ultimately, resulted in the identification of 24 genes that appear to be required for cell fusion between CATs (25). This gene set included *ada-3*, for which only co-segregation data were used as confirmation of the association between the phenotype and the lack of *ada-3* (see Discussion), and which we show here to have been misclassified as a cell fusion gene. As part of their study, and in addition to the candidate cell fusion-related genes, Fu et al. also reported that while numerous KO strains exhibited a flat conidiation phenotype, they could not be rescued by introduction of the WT copy of the corresponding gene. We surmised that for at least some of these strains, the phenotype may be due to the ~30-Kb deletion.

**Fig 3:**
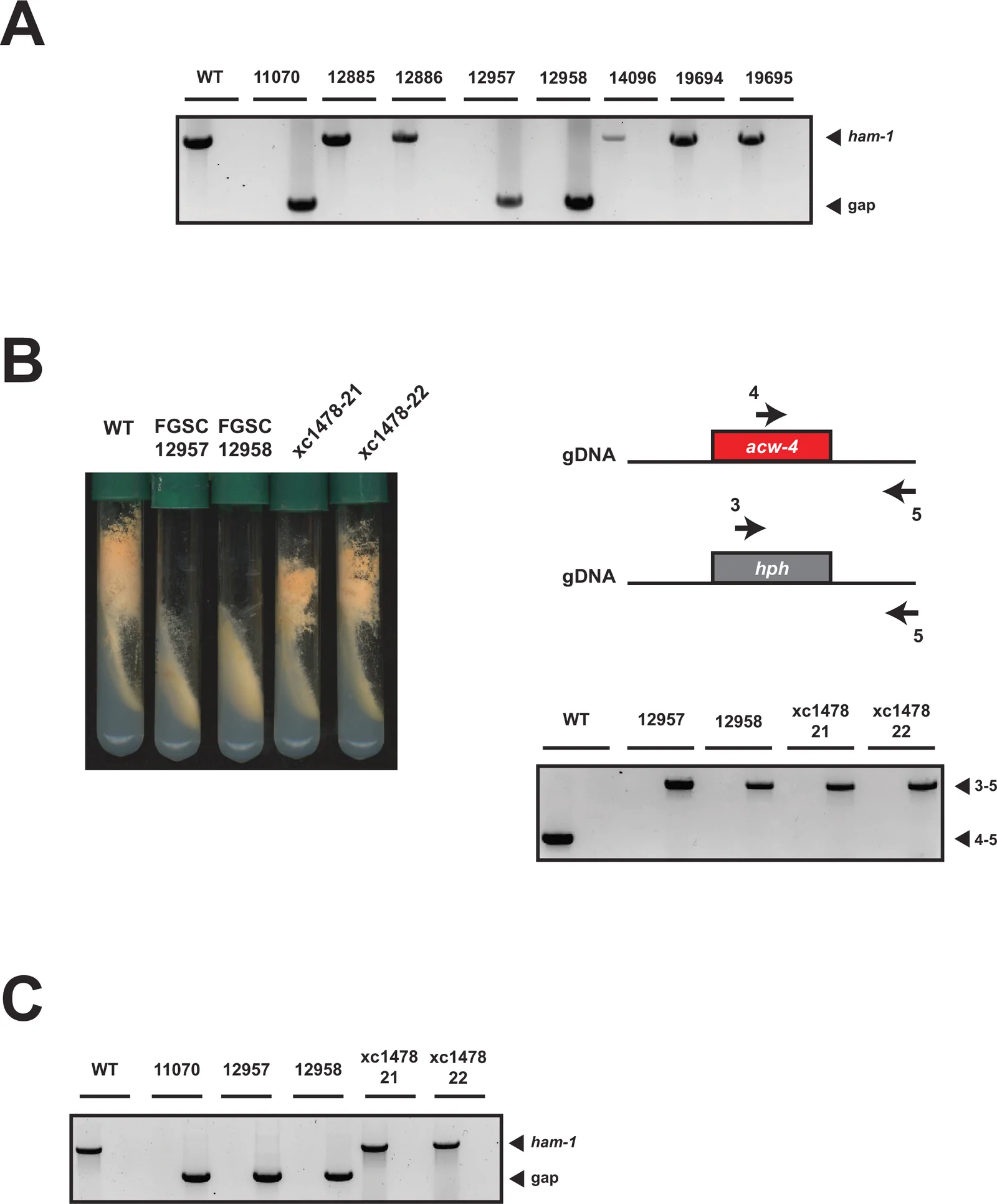
Strains FGSC 12957 and 12958 also harbor the ~30-Kb deletion. (**A**) PCR analysis to evaluate the presence of *so/ham-1* or the ~30-Kb gap in the genome of the strains listed. To detect *so/ham-1*, primers within its coding region were used. To evaluate the presence of the deletion, primers flanking the ~30-Kb gap were used such that under normal conditions (i.e., no deletion), the size of the DNA fragment between the primers (~30 Kb) would be too large to be amplified with the PCR settings used. Conversely, if the deletion is present, a PCR product could be obtained under the conditions used. We used a nested PCR strategy to detect the gap (Table 1). Note that a homokaryotic strain can either be positive for *so/ham-1* or harbor the deletion, but not both. As such, even though all strains were tested for both targets, only one can be detected. (**B**) (Left) Phenotypic analysis. WT, FGSC 12957, FGSC 12958, and two WT-like Δ*acw-4* progeny derived from crossing WT with 12957 (xc1478, clones 21 and 22) were imaged after culture on slants of VMM for 7 days at 25°C under constant light. (Right) Schematic diagram and genotyping of the strains on the left, to confirm the Δ*acw-4* status (and concomitant replacement of *acw-4* with the *hph* cassette) of the 12957, 12958, and xc1478 strains. Note that even though both reactions were tested per homokaryotic strain, each strain can only be positive for either the WT gene or the KO cassette, but not both. (**C**) PCR analysis to evaluate the presence of *so/ham-1* or the ~30-Kb gap in the genome of the strains shown in (**B**), with 11070 as reference for the deletion. Note that a homokaryotic strain can either be positive for *so/ham-1* or harbor the deletion, but not both. All primer sequences are shown in Table 1.

To test this, we decided to evaluate the presence of the ~30-Kb deletion in the KO strains that exhibit a flat conidiation phenotype but for which Fu et al. (25) reported that complementation was unsuccessful, that is, that introduction of a WT copy of the target gene did not revert the mutant phenotype. These corresponded to seven strains, representing four different genes. Interestingly, we found that two of these strains, FGSC 12957 and 12958, which are the two deposited KO strains for *NCU09263* (*acw-4*), harbor the ~30-Kb deletion (Fig. 3A) and, correspondingly, lack the genes in the deleted region in addition to the intended KO gene, *acw-4*. This suggested to us that the phenotype exhibited by 12957/12958 is unrelated to the absence of *acw-4* and might, instead, be due to the ~30-Kb deletion (and the corresponding absence of *so*/*ham-1*). Consistent with this idea, we were able to obtain, via crossing, Δ*acw-4* clones that exhibit a WT phenotype (Fig. 3B). These WT-like Δ*acw-4* clones do not harbor the ~30-Kb deletion, while the Δ*acw-4* clones that exhibit an ADA phenotype do (Fig. 3C), similar to the situation with 11070 (see Fig. 1A and B).

The other five strains tested did not harbor the ~30-Kb deletion. Given, however, that previous evidence has shown that mutations in the *so*/*ham-1* gene can arise as cheater variants during vegetative propagation [see (39) and Discussion], we surmised that these strains might nevertheless contain mutations within this gene that would be responsible for their phenotype. We found, however, that this was not the case; sequencing of the *so/ham-1* locus revealed no mutations in these KO strains.

**Fig 4:**
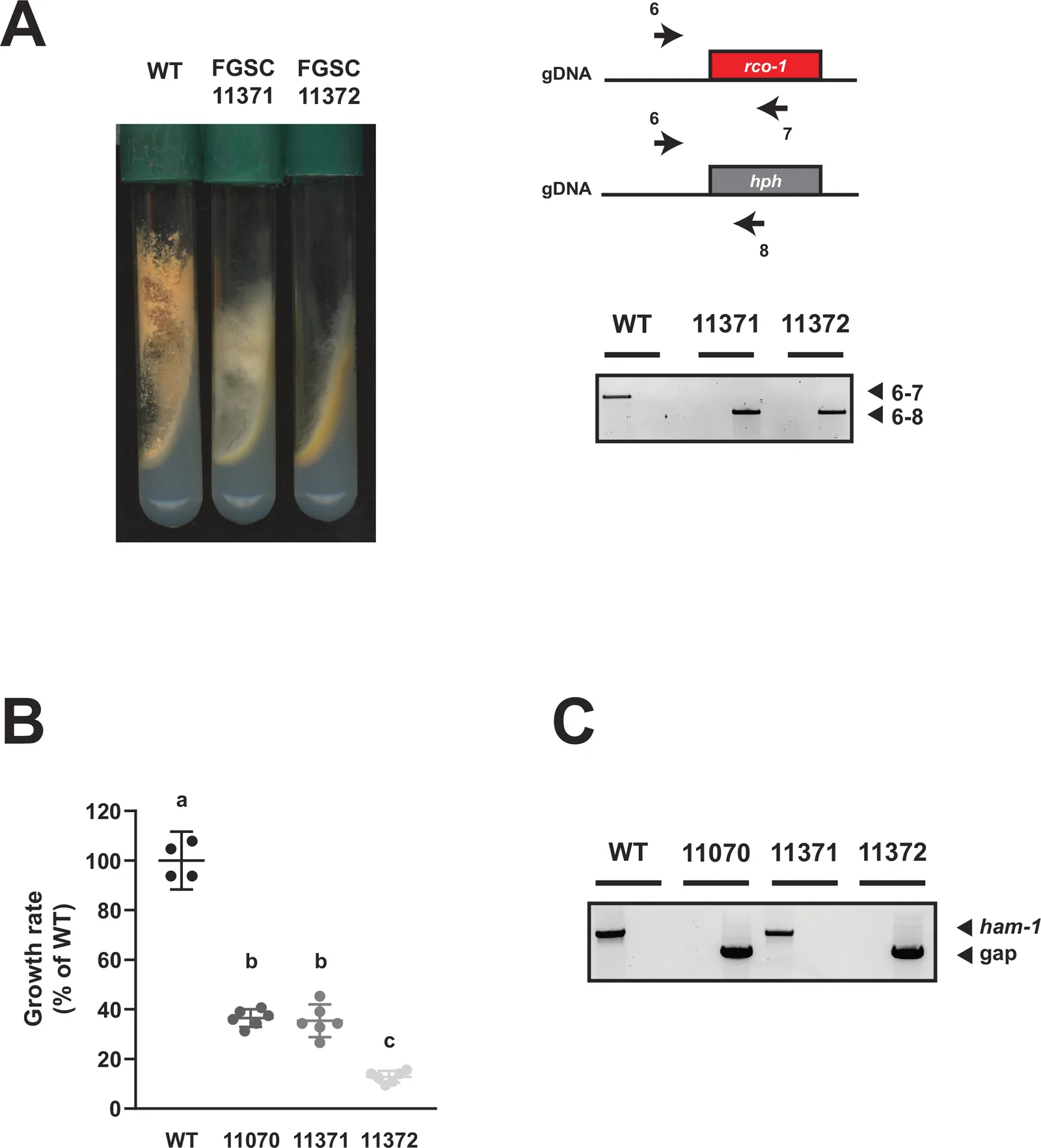
FGSC 11372, an *rco-1* KO strain, harbors the ~30-Kb deletion. (**A**) (Left) Phenotypic analysis of WT and the two *rco-1* KO strains generated as part of Neurospora Functional Genomics Project, FGSC 11371 and 11372. The strains were imaged after culture on slants of VMM for 7 days at 25°C under constant light. (Right) Schematic diagram and genotyping of the strains on the left, to confirm the Δ*rco-1* status (and concomitant replacement of *rco-1* with the *hph* cassette) of the 11371 and 11372 strains. Note that even though both reactions were tested per homokaryotic strain, each strain can only be positive for either the WT gene or the KO cassette, but not both. (**B**) Growth rate assay. Conidia from the strains listed were inoculated on race tubes containing VMM and were then placed under constant light conditions at 25°C. The growth front was marked on the tubes every 24 h, and the distance between the marks was then used to calculate the linear growth rate per day. Dots represent data from four to six independent biological replicates. Shown are the mean ± 95% confidence intervals. Analysis of variance, F (3, 18) = 257.5, *P* < 0.0001. Different letters indicate statistically significant differences between groups (Tukey's Honest Significant Difference , *P* < 0.05). All statistical analyses were performed using GraphPad Prism v. 9.5.1 (733). (**C**) PCR analysis to evaluate the presence of *so/ham-1* or the ~30-Kb gap in the genome of the strains shown in (**B**). Note that a homokaryotic strain can either be positive for *so/ham-1* or harbor the deletion, but not both. All primer sequences are shown in Table 1.

Fu et al. (25) also reported *regulator of conidiation 1* (*rco-1*), ortholog of the *Saccharomyces cerevisiae TUP1* gene, to be a candidate cell fusion gene. This supported an earlier study that suggested a role for *rco-1* in hyphal fusion in *N. crassa* (40). Consistent with this, the *rco-1* mutant exhibits numerous developmental defects, including an altered conidiation pattern and female sterility (41), typical of many cell fusion mutants (23). More recently, our group reported a role for RCO-1 in modulating circadian gene expression and metabolic compensation in *N. crassa* (42). During the course of that study, we noticed that the two deposited KO strains for this gene, 11371 (Δ*NCU06205*, A) and 11372 (Δ*NCU06205*, a), generated as part of the Neurospora KO project, exhibit different overt phenotypes, particularly in aerial hyphae development (Fig. 4A). Furthermore, while both strains display reduced hyphal growth rates compared to the WT, a finding consistent with previous studies of the Δ*rco-1* strain (41), the phenotype is significantly more severe in 11372. Indeed, the observed mean growth rate (95% CI) of 11371 was 35.4% (28.8–42.03) relative to WT, while that of 11372 was 12.8% (10.35–15.17) (Fig. 4B). Given that we noticed this discrepancy at the same time as we were evaluating strains for the ~30-Kb deletion, and that Δ*rco-1* has been reported as a cell fusion-defective mutant, we decided to test whether any of these Δ*rco-1* strains harbored the ~30-Kb deletion. We found that 11372 does indeed harbor the ~30-Kb deletion (Fig. 4C), meaning that while this strain is an *rco-1* deletion mutant (Fig. 4A), it also lacks the other genes in the deleted fragment, including *so/ham-1*. These genetic alterations in 11372 (i.e., both the lack of *rco-1* and the presence of the ~30-Kb deletion) appear to have a multiplicative effect on growth rate. Indeed, the observed mean growth rate (95% CI) of 11070 was 36.4% (32.92–40) relative to WT, and the growth rate defect of the 11372 double mutant equals the product of the single-deletion effects observed in 11371 and 11070 (Fig. 4B).

Together, these results show that the ~30-Kb deletion originally found in 11070 is present in multiple strains of the Neurospora KO collection, and highlight that secondary mutations and genomic rearrangements may be present in multiple strains in the collection and be responsible for various reported phenotypes, many of which might have been attributed to the deletion of an unrelated gene.

## DISCUSSION

In this study, we report that multiple strains of the *Neurospora crassa* KO collection that exhibit an abnormal phenotype typical of many cell fusion-defective mutants, share a common secondary genetic alteration, namely, a ~30-Kb deletion affecting three genes. Our data suggest that it is this deletion—and not the absence of the intended target gene—that is responsible for the pleiotropic phenotype in these strains. Our results highlight the importance of proper functional validation of strains from the *N. crassa* KO collection—and from mutant collections in general—and suggest that conclusions of studies that have used the strains herein reported, or others with similar phenotypes, may need to be reevaluated.

A classical and powerful approach to study gene function is to create loss-of-function mutants and then observe the resulting phenotypes. As part of the Neurospora Functional Genomics Project (9), deletion strains for the vast majority of this organism’s predicted ~10,000 protein-coding genes have been created and made available to the community. These strains represent an invaluable resource, and have been used by numerous laboratories for both focused and genome-wide screens [see, for instance, references (10, 12 – 16, 43 – 45)], providing key insights into gene function in this model system.

In the first study describing the methodology for high-throughput generation of loss-of-function deletion strains in *N. crassa*, Colot et al. (10) reported the creation of deletion strains for 103 genes that encode for predicted transcription factors. The authors then subjected the strains to phenotypic analysis, and reported that ~40% of the deletion mutants exhibit defects in basal hyphal extension, vegetative development, and/or sexual development. Nine strains were found to display defects in all three of these traits, and seven of them were thus termed “*all development altered*” (*ada-1* to *ada-7*). While Southern blots were used to ensure correct and unique insertion of the knockout cassette, and to confirm the homokaryotic status of the mutant, no further analyses were done to determine whether the observed phenotypes were indeed due to the deletion of the target gene.

As part of a routine genetic study, we found that the pleiotropic phenotype exhibited by the deposited KO strain for *all development altered (ada)-3* (FGSC 11070; Δ*NCU02896*) was not due to the deletion of *ada-3*. Indeed, we found that the ADA phenotype did not co-segregate with the deletion cassette—a finding consistent with a previous report (46)—which suggested that the phenotype was the result of a secondary genetic alteration (Fig. 1). To identify the mutation responsible for the phenotype in this strain, we used whole-genome sequencing, which revealed a ~30-Kb deletion in LG I that affects three genes (Fig. 2A). We observed that deletion of only one of these genes, *so*/*ham-1,* results in the same abnormal phenotype reported for FGSC 11070 (Fig. 2B). We performed a complementation assay with a WT copy of *so*/*ham-1*, and confirmed that the phenotype in 11070 was indeed a result of the deletion of *so*/*ham-1* and is unrelated to the lack of *ada*-3 (Fig. 1 and 2). Given that deletion of *NCU02896* does not result in a strain displaying an ADA phenotype, renaming *ada-3* should be considered.

The *ada-3* gene has previously been suggested to play a role in cell fusion. This was based on a study by Fu et al. (25), who set out to find cell fusion-related genes in *N. crassa*. For this, they screened the KO collection for strains that exhibited a flat conidiation pattern and a defect or delay in the production of protoperithecia, a phenotype common to many cell fusion mutants (23), including *so*/*ham-1*, which is known to play a role in cell fusion in *N. crassa* (38). The screening was followed by segregation, complementation, and/or functional assays, which, ultimately, resulted in the identification of 24 genes that appeared to be required for cell fusion between conidial anastomosis tubes in Neurospora; this gene set included *ada-3* (25). For *ada-3*, however, the authors relied solely on co-segregation data to determine that the observed phenotype was due to the absence of this gene. Indeed, given that the *ada-3* locus (*NCU02896*) lies close to the herein identified ~30-Kb deletion in LG I, it is likely that the co-segregation data—particularly if based on scoring a relatively small number of progeny when back-crossing FGSC 11070— might have suggested to the authors that the phenotype and the deletion cassette co-segregate, which would have led Fu et al. (25) to classify *ada-3* as a cell fusion gene. As mentioned above, however, we report here that deletion of *ada-3* does not result in such phenotype (Fig. 1), and that the pleiotropic phenotype reported by Fu et al. (25) for the *ada-3* deletion strain (FGSC 11070) is instead due to the concomitant deletion of *so*/*ham-1* in the Δ*ada-3* background. Indeed, deletion of *so*/*ham-1* on its own results in that phenotype—while deletion of *ada-3* does not—and introduction of a WT copy of *so*/*ham-1* reverts the ADA phenotype of the Δ*ada-3* strain (Fig. 1 and 2). As such, our study shows that *ada-3*, on its own, appears to play no role in cell fusion, or at least not one that would result in the overt phenotype that Fu et al. (25) attributed to its deletion.

As part of their screening, Fu et al. (25) also reported seven KO strains for which re-introduction of a WT copy of the target gene could not rescue the mutant phenotype of interest (i.e., a flat conidiation pattern and a defect or delay in the production of protoperithecia). Given that this phenotype is consistent with the ADA phenotype, we explored whether at least some of these strains harbored the ~30-Kb deletion as a secondary mutation, such that the lack of *so*/*ham-1* would be responsible for their observed phenotype. We indeed found two such strains, FGSC 12957/12958, which, while they both lack the target gene *NCU09263*/*acw-4*, also harbor the ~30-Kb deletion. Via crossing, we were able to obtain Δ*acw-4* strains that either had the ~30-Kb deletion or did not feature such deletion; the former displayed the ADA phenotype, while the latter did not, suggesting that the ADA phenotype in these strains is due to the ~30-Kb deletion and further confirming that it is unrelated to the absence of *acw-4*. Maddi et al. (47) reported that while they did observe co-segregation of the mutant phenotype with the deletion cassette in the Δ*acw-4* strain, the phenotype was not reverted by introduction of a WT copy of *acw-4*, which supports the idea that there is a secondary mutation responsible for the phenotype (38). Our data suggest that this secondary mutation corresponds to the ~30-Kb deletion, specifically to the absence of *so*/*ham-1*. Importantly, the ~30-Kb deletion in the Δ*acw-4* strain would explain the results reported by Maddi et al. (47): given that the deletion lies close to *acw-4* locus in LG I, one would expect to see moderate co-segregation of the phenotype with the deletion cassette but no complementation when transforming with a WT copy of *acw-4*. The other five strains analyzed based on the study by Fu et al (25) do not harbor the ~30-Kb deletion. Given that a previous study reported that mutations in *so*/*ham-1* can arise as cheater variants during vegetative propagation, resulting in the same pleiotropic phenotype exhibited by these strains (39), we tested whether they contained mutations in this gene. No mutations, however, were found. Additional studies of these strains are needed to uncover the molecular mechanisms underlying their phenotype.

Among the 24 genes that Fu et al. (25) reported as involved in cell fusion, was *regulator of conidiation 1* (*rco-1*) (41). This gene had previously been reported to play a role in cell fusion in Neurospora (40), which lent support to the screening. During a study in our laboratory on the role of RCO-1 in circadian gene expression and metabolic compensation in *N. crassa* (42), we noticed that the two deposited KO strains for this gene, FGSC 11371 and 11372, exhibit different phenotypes (Fig. 4A). We found that, in addition to lacking *rco-1*, 11372 harbors the ~30-Kb deletion. Interestingly, while it has previously been reported that deletion of *rco-1* results in a reduced growth rate (41), we noticed that the defect is even more dramatic in 11372 (Fig. 4B). Mutations in *so*/*ham-1* are known to affect growth rate (37), and the phenotype exhibited by 11372 appears to be the result of the compounded effect of the lack of both *rco-1* and *so*/*ham-1*. This multiplicative effect suggests that these loci may have an independent effect on growth rate in *N. crassa* (48).

Given that FGSC 11372 has a secondary mutation that affects its overt phenotype, it would thus be inappropriate for functional studies of *rco-1*: any result would be confounded by the fact that, in addition to lacking *rco-1*, this strain is also a *so*/*ham-1* mutant. As such, the conclusions of functional studies of *rco-1* based on 11372 may need to be reevaluated [see (13, 16, 25, 40, 49 – 51)]. Of particular notice among these studies is the report by Carrillo et al. (13), who characterized the growth and developmental phenotypes of loss-of-function deletion mutants of a large set of predicted transcription factor genes in *N. crassa*, including the transcriptional co-repressor RCO-1. In this work, it was reported that ~40% of viable mutants had a growth rate significantly slower than WT, and that deletion of *rco-1* resulted in the lowest growth rate observed in the study, at ~10% of the WT rate. This is significantly different from the rate observed for Δ*rco-1* in previous studies (41) and in our laboratory (Fig. 4B) (~30% of WT), but is similar to the rate we observed for 11372 (Fig. 4B), which, as reported here, lacks both *rco-1* and *so*/*ham-1*; indeed, the authors used 11372 for their analysis of *rco-1* and, thus, the classification of Δ*rco-1* as the slowest-growing mutant in this data set is likely unwarranted. Similarly, this study also identified 92 mutant strains with a defect in aerial hyphae height and/or conidia production, and reported that the Δ*rco-1* strain exhibited the shortest aerial hyphae among all tested mutants. This, again, is likely a consequence of the additional mutation in this strain, as mutations in *so*/*ham-1* are also known to affect aerial hyphae development (38).

Secondary mutations exist in multiple strains of the *N. crassa* KO collection (25, 26), and many of these appear to result in an ADA phenotype. Interestingly, it has long been known that spontaneous mutations affecting growth rate and morphology—resulting in phenotypes similar to that of *so*/*ham-1*—are common and may arise in any strain, even in WT back-crosses (24). Indeed, such mutants are frequently found in all Neurospora laboratories. The reasons behind this are unclear, but some recent data shed light on how these may be propagated, and allow us to speculate about how they might have contaminated the *N. crassa* KO collection. In two studies, involving experimental evolution, whole-genome sequencing, and various competition assays, the Aanen group (39, 52) proposed a model to explain how cheater genotypes, which appear to involve cell fusion-defective mutants —including *so*/*ham-1* mutants— may be propagated during clonal growth. Maintaining live cultures by serial transfers may favor the appearance of such cheater lineages and, ultimately, reduce overall performance (conidial spore yield). Briefly, the authors proposed that upon a *de novo* cell fusion cheater mutation, the mutant nuclei will have increased representation in the aerial hyphae (and the resulting conidia) by virtue of the reduced chance of cheaters to participate in hyphal fusion. Upon clonal propagation and germination, a fraction of these mutants will fuse with WT or heterokaryotic mycelia, thus forming new heterokaryons. Importantly, while the mutants are unable to initiate fusion, WT hyphae can fuse to them (39). Fusion will give cheaters access to a well-connected mycelia, with efficient resource distribution and utilization, and will allow them to propagate effectively. When cheaters reach higher frequencies, however, the culture becomes increasingly fragmented, and cheater mycelia will remain largely unconnected from WT hyphae, which will ultimately reduce the total spore yield of the culture. The spread of such female-sterile cheaters is consistent with the early observation made by Westergaard and Hirsch that cultures of Neurospora commonly become female sterile after extended periods of vegetative propagation (53). Culture degeneration, with loss of female fertility, has also been reported in *Fusarium* species (54).

As such, the results of the Aanen group suggest that many of the spontaneous morphological mutants frequently found in Neurospora labs may be the result of unintended selection of cell fusion-defective cheater variants during routine propagation (39). In this context, it is conceivable that among the conidia in a spore suspension from a seemingly WT serially propagated culture, there could be multiple cheater mutant nuclei. Indeed, early reports suggested that cultures of strains typically used as WT in Neurospora (of the Oak Ridge background) may commonly become heterokaryotic for mutations that result in female sterility and *soft*-like phenotypes (24, 55). It is thus possible that these nuclei, in the conidial suspension, are transformed and selected for when generating, for instance, KO strains, such as the ones described in our study. In this scenario, it could be proposed that strains FGSC 11070, 12957/12958, and 11372 might have been derived from transformation of mutant conidia from the same parental strain. Efforts to track down this information, however, were unsuccessful, as the Neurospora KO project was completed several years ago. While all of these mutants appear to have been generated in the same laboratory (Dunlap lab, Dartmouth University), the group confirmed they were done on different transformation plates/different batches (J. C. Dunlap, personal communication), but the source of the conidia (i.e., the exact batch used) is unknown. Alternatively, although intuitively less likely, the existence of this mutation in these different KO strains might have been due to independent events; as suggested by Grum-Grzhimaylo et al. (39), different types of mutations in *so*/*ham-1*—of which the ~30-Kb deletion could be an example—might be common as cheaters, and could have appeared independently in different clonally propagated WT cultures used as a source of conidia for transformation experiments for the Neurospora KO project. Indeed, Grum-Grzhimaylo et al. (39) found that 75% (6/8) of the cheater lines analyzed had acquired mutations in the *so*/*ham-1* gene. In addition, ~50 years ago, a spontaneous female sterility mutant, female sterile-n (*fs-n*), was obtained via conidial isolation of an established WT laboratory strain (74-OR23-1 A, FGSC 987), where it occurred in a heterokaryotic condition (55). The *fs-n* strain was more recently subjected to whole-genome sequencing and found to contain a + 1:A frameshift mutation in *so*/*ham-1* (56).

In summary, we report here that multiple strains of the *N. crassa* KO collection harbor a ~30-Kb deletion in LG I, and that this deletion might have affected the conclusions of various studies. We found four strains in this collection with such a secondary mutation affecting *so*/*ham-1*, but given that our analysis was neither systematic nor comprehensive, the extent of the issue is currently unknown but likely affects many more strains, particularly if other types of mutations in *so*/*ham-1* are considered. Our data should serve as a cautionary note regarding the use of strains from the *N. crassa* KO collection—and mutant collections in general—and highlight that standard validation assays should always be performed to confirm that a phenotype in a Neurospora KO strain is indeed due to the mutation of the target gene. These include not only strain validation by PCR and segregation assays but, ideally, also complementation. In addition, it is important to consider proper strain storage and handling (57). Users should place primary stocks into suspended animation and minimize their vegetative transfer, to avoid the acquisition of mutations. Furthermore, users would be best served by occasionally replacing laboratory stocks with those from the FGSC and by purifying strains of interest (e.g., by crossing and ascospore isolation). While whole-genome sequencing could be proposed for routine strain and sample verification, the associated costs and the paucity of suitable and easily accessible bioinformatics tools create a barrier for such an approach (17), further supporting the implementation of the simple assays and procedures described above.

Secondary mutations are widespread in the *N. crassa* KO collection, and many of these result in cell fusion-defective phenotypes, so researchers should consider the *so*/*ham-1* status of KO strains that exhibit such a phenotype before performing any assays. With careful validation of the results, mutant collections, including the *N*. *crassa* KO collection, will continue to serve as incredibly useful resources for the community.

## Data Availability

Sequencing reads are available from the NCBI BioProject database under accession number PRJNA971561.

## References

[1] Roche CM , Loros JJ , McCluskey K , Glass NL . 2014. Neurospora crassa: looking back and looking forward at a model microbe. Am J Bot 101:2022–2035. doi:10.3732/ajb.1400377 25480699

[2] Davis RH . 2000. Neurospora: contributions of a model organism. Oxford University Press.

[3] Galagan JE , Calvo SE , Borkovich KA , Selker EU , Read ND , Jaffe D , FitzHugh W , Ma L-J , Smirnov S , Purcell S , Rehman B , Elkins T , Engels R , Wang S , Nielsen CB , Butler J , Endrizzi M , Qui D , Ianakiev P , Bell-Pedersen D , Nelson MA , Werner-Washburne M , Selitrennikoff CP , Kinsey JA , Braun EL , Zelter A , Schulte U , Kothe GO , Jedd G , Mewes W , Staben C , Marcotte E , Greenberg D , Roy A , Foley K , Naylor J , Stange-Thomann N , Barrett R , Gnerre S , Kamal M , Kamvysselis M , Mauceli E , Bielke C , Rudd S , Frishman D , Krystofova S , Rasmussen C , Metzenberg RL , Perkins DD , Kroken S , Cogoni C , Macino G , Catcheside D , Li W , Pratt RJ , Osmani SA , DeSouza CPC , Glass L , Orbach MJ , Berglund JA , Voelker R , Yarden O , Plamann M , Seiler S , Dunlap J , Radford A , Aramayo R , Natvig DO , Alex LA , Mannhaupt G , Ebbole DJ , Freitag M , Paulsen I , Sachs MS , Lander ES , Nusbaum C , Birren B . 2003. The genome sequence of the filamentous fungus Neurospora crassa. Nature 422:859–868. doi:10.1038/nature01554 12712197

[4] Tabilo-Agurto C , Del Rio-Pinilla V , Eltit-Villarroel V , Goity A , Muñoz-Guzmán F , Larrondo LF . 2023. Developing a temperature-inducible transcriptional rheostat in Neurospora crassa. mBio 14:e0329122. doi:10.1128/mbio.03291-22 36744948 PMC9973361

[5] Matsu-Ura T , Baek M , Kwon J , Hong C . 2015. Efficient gene editing in Neurospora crassa with CRISPR technology. Fungal Biol Biotechnol 2:4. doi:10.1186/s40694-015-0015-1 28955455 PMC5611662

[6] He L , Guo W , Li J , Meng Y , Wang Y , Lou H , He Q . 2020. Two dominant selectable markers for genetic manipulation in Neurospora crassa. Curr Genet 66:835–847. doi:10.1007/s00294-020-01063-1 32152733

[7] Perkins DD , Davis RH . 2000. Neurospora at the millennium. Fungal Genet Biol 31:153–167. doi:10.1006/fgbi.2000.1248 11273678

[8] Borkovich KA , Alex LA , Yarden O , Freitag M , Turner GE , Read ND , Seiler S , Bell-Pedersen D , Paietta J , Plesofsky N , Plamann M , Goodrich-Tanrikulu M , Schulte U , Mannhaupt G , Nargang FE , Radford A , Selitrennikoff C , Galagan JE , Dunlap JC , Loros JJ , Catcheside D , Inoue H , Aramayo R , Polymenis M , Selker EU , Sachs MS , Marzluf GA , Paulsen I , Davis R , Ebbole DJ , Zelter A , Kalkman ER , O’Rourke R , Bowring F , Yeadon J , Ishii C , Suzuki K , Sakai W , Pratt R . 2004. Lessons from the genome sequence of Neurospora crassa: tracing the path from genomic blueprint to multicellular organism. Microbiol Mol Biol Rev 68:1–108. doi:10.1128/MMBR.68.1.1-108.2004 15007097 PMC362109

[9] Dunlap JC , Borkovich KA , Henn MR , Turner GE , Sachs MS , Glass NL , McCluskey K , Plamann M , Galagan JE , Birren BW , Weiss RL , Townsend JP , Loros JJ , Nelson MA , Lambreghts R , Colot HV , Park G , Collopy P , Ringelberg C , Crew C , Litvinkova L , DeCaprio D , Hood HM , Curilla S , Shi M , Crawford M , Koerhsen M , Montgomery P , Larson L , Pearson M , Kasuga T , Tian C , Baştürkmen M , Altamirano L , Xu J . 2007. Enabling a community to dissect an organism: overview of the *Neurospora* functional genomics project, p 49–96. In Advances in Genetics. Elsevier. doi:10.1016/S0065-2660(06)57002-6 PMC3673015 17352902

[10] Colot HV , Park G , Turner GE , Ringelberg C , Crew CM , Litvinkova L , Weiss RL , Borkovich KA , Dunlap JC . 2006. A high-throughput gene knockout procedure for Neurospora reveals functions for multiple transcription factors. Proc Natl Acad Sci U S A 103:10352–10357. doi:10.1073/pnas.0601456103 16801547 PMC1482798

[11] McCluskey K , Wiest A , Plamann M . 2010. The fungal Genetics stock center: a repository for 50 years of fungal genetics research. J Biosci 35:119–126. doi:10.1007/s12038-010-0014-6 20413916

[12] Cabrera IE , Pacentine IV , Lim A , Guerrero N , Krystofova S , Li L , Michkov AV , Servin JA , Ahrendt SR , Carrillo AJ , Davidson LM , Barsoum AH , Cao J , Castillo R , Chen W-C , Dinkchian A , Kim S , Kitada SM , Lai TH , Mach A , Malekyan C , Moua TR , Torres CR , Yamamoto A , Borkovich KA . 2015. Global analysis of predicted G protein-coupled receptor genes in the filamentous fungus, Neurospora crassa. G3 (Bethesda) 5:2729–2743. doi:10.1534/g3.115.020974 26464358 PMC4683645

[13] Carrillo AJ , Schacht P , Cabrera IE , Blahut J , Prudhomme L , Dietrich S , Bekman T , Mei J , Carrera C , Chen V , Clark I , Fierro G , Ganzen L , Orellana J , Wise S , Yang K , Zhong H , Borkovich KA . 2017. Functional profiling of transcription factor genes in Neurospora crassa. G3 (Bethesda) 7:2945–2956. doi:10.1534/g3.117.043331 28696922 PMC5592922

[14] Ghosh A , Servin JA , Park G , Borkovich KA . 2014. Global analysis of serine/threonine and tyrosine protein phosphatase catalytic subunit genes in Neurospora crassa reveals interplay between phosphatases and the p38 mitogen-activated protein kinase. G3 (Bethesda) 4:349–365. doi:10.1534/g3.113.008813 24347630 PMC3931568

[15] Park G , Servin JA , Turner GE , Altamirano L , Colot HV , Collopy P , Litvinkova L , Li L , Jones CA , Diala F-G , Dunlap JC , Borkovich KA . 2011. Global analysis of serine-threonine protein kinase genes in Neurospora crassa▿. Eukaryot Cell 10:1553–1564. doi:10.1128/EC.05140-11 21965514 PMC3209061

[16] Carrillo AJ , Cabrera IE , Spasojevic MJ , Schacht P , Stajich JE , Borkovich KA . 2020. Clustering analysis of large-scale phenotypic data in the model filamentous fungus Neurospora crassa. BMC Genomics 21:755. doi:10.1186/s12864-020-07131-7 33138786 PMC7607824

[17] Gallegos JE , Hayrynen S , Adames NR , Peccoud J . 2020. Challenges and opportunities for strain verification by whole-genome sequencing. Sci Rep 10:5873. doi:10.1038/s41598-020-62364-6 32245992 PMC7125075

[18] Ajjawi I , Lu Y , Savage LJ , Bell SM , Last RL . 2010. Large-scale reverse genetics in Arabidopsis: case studies from the chloroplast 2010 project. Plant Physiol 152:529–540. doi:10.1104/pp.109.148494 19906890 PMC2815874

[19] Sarin S , Bertrand V , Bigelow H , Boyanov A , Doitsidou M , Poole RJ , Narula S , Hobert O . 2010. Analysis of multiple ethyl methanesulfonate-mutagenized caenorhabditis elegans strains by whole-genome sequencing. Genetics 185:417–430. doi:10.1534/genetics.110.116319 20439776 PMC2881126

[20] Teng X , Dayhoff-Brannigan M , Cheng W-C , Gilbert CE , Sing CN , Diny NL , Wheelan SJ , Dunham MJ , Boeke JD , Pineda FJ , Hardwick JM . 2013. Genome-wide consequences of deleting any single gene. Mol Cell 52:485–494. doi:10.1016/j.molcel.2013.09.026 24211263 PMC3975072

[21] Puddu F , Herzog M , Selivanova A , Wang S , Zhu J , Klein-Lavi S , Gordon M , Meirman R , Millan-Zambrano G , Ayestaran I , Salguero I , Sharan R , Li R , Kupiec M , Jackson SP . 2019. Genome architecture and stability in the S. cerevisiae knockout collection. Nature 573:416–420. doi:10.1038/s41586-019-1549-9 31511699 PMC6774800

[22] Parker DJ , Demetci P , Li G-W . 2019. Rapid accumulation of motility-activating mutations in resting liquid culture of Escherichia coli. J Bacteriol 201:e00259-19. doi:10.1128/JB.00259-19 31285239 PMC6755740

[23] Fischer MS , Glass NL . 2019. Communicate and fuse: how filamentous fungi establish and maintain an interconnected mycelial network. Front Microbiol 10:619. doi:10.3389/fmicb.2019.00619 31001214 PMC6455062

[24] Kafer E . 1982. Improved backcrossed strains giving consistent map distances. Fungal Genetics Reports 29:41–44. doi:10.4148/1941-4765.1645

[25] Fu C , Iyer P , Herkal A , Abdullah J , Stout A , Free SJ . 2011. Identification and characterization of genes required for cell-to-cell fusion in Neurospora crassa. Eukaryot Cell 10:1100–1109. doi:10.1128/EC.05003-11 21666072 PMC3165452

[26] Chinnici JL , Fu C , Caccamise LM , Arnold JW , Free SJ , Pöggeler S . 2014. Neurospora crassa female development requires the PACC and other signal transduction pathways, transcription factors, chromatin remodeling, cell-to-cell fusion, and autophagy. PLoS ONE 9:e110603. doi:10.1371/journal.pone.0110603 25333968 PMC4204872

[27] Davis RH , de FJ . 1970. [4] Genetic and Microbiological research techniques for *Neurospora crassa* , p 79–143. In Methods in Enzymology. Academic Press.

[28] Vogel HJ . 1956. A convenient growth medium for Neurospora. Microb Genet Bull 13:42–43.

[29] Ryan FJ , Beadle GW , Tatum EL . 1943. The tube method of measuring the growth rate of Neurospora. Am J Bot 30:784–799. doi:10.1002/j.1537-2197.1943.tb10332.x

[30] Westergaard M , Mitchell HK . 1947. Neurospora V. A synthetic medium favoring sexual reproduction. Am J Bot 34:573–577. doi:10.1002/j.1537-2197.1947.tb13032.x

[31] Montenegro-Montero A . 2014. BZIP transcription factors and transcriptional regulatory networks in the Neurospora circadian system Ph.D, Pontificia Universidad Católica de Chile, Santiago, Chile

[32] Li H , Durbin R . 2009. Fast and accurate short read alignment with Burrows-Wheeler transform. Bioinformatics Oxf Engl 25:1754–1760. doi:10.1093/bioinformatics/btp324 PMC2705234 19451168

[33] Li H , Handsaker B , Wysoker A , Fennell T , Ruan J , Homer N , Marth G , Abecasis G , Durbin R , 1000 Genome project data processing subgroup . 2009. The sequence alignment/map format and SAMtools. Bioinforma Oxf Engl 25:2078–2079. doi:10.1093/bioinformatics/btp352 PMC2723002 19505943

[34] Robinson JT , Thorvaldsdóttir H , Winckler W , Guttman M , Lander ES , Getz G , Mesirov JP . 2011. Integrative genomics viewer. Nat Biotechnol 29:24–26. doi:10.1038/nbt.1754 21221095 PMC3346182

[35] Margolin BS , Freitag M , Selker EU . 1997. Improved plasmids for gene targeting at the his-3 locus of Neurospora crassa by electroporation. Fungal Genet Rep 44:34–36. doi:10.4148/1941-4765.1281

[36] Weirauch MT , Yang A , Albu M , Cote AG , Montenegro-Montero A , Drewe P , Najafabadi HS , Lambert SA , Mann I , Cook K , Zheng H , Goity A , van Bakel H , Lozano J-C , Galli M , Lewsey MG , Huang E , Mukherjee T , Chen X , Reece-Hoyes JS , Govindarajan S , Shaulsky G , Walhout AJM , Bouget F-Y , Ratsch G , Larrondo LF , Ecker JR , Hughes TR . 2014. Determination and inference of eukaryotic transcription factor sequence specificity. Cell 158:1431–1443. doi:10.1016/j.cell.2014.08.009 25215497 PMC4163041

[37] Wilson JF , Dempsey JA . 1999. A hyphal fusion mutant in Neurospora crassa. Fungal Genet Rep 46:25–30. doi:10.4148/1941-4765.1240

[38] Fleissner A , Sarkar S , Jacobson DJ , Roca MG , Read ND , Glass NL . 2005. The so locus is required for vegetative cell fusion and postfertilization events in Neurospora crassa. Eukaryot Cell 4:920–930. doi:10.1128/EC.4.5.920-930.2005 15879526 PMC1140088

[39] Grum-Grzhimaylo AA , Bastiaans E , van den Heuvel J , Berenguer Millanes C , Debets AJM , Aanen DK . 2021. Somatic deficiency causes reproductive parasitism in a fungus. Nat Commun 12:783. doi:10.1038/s41467-021-21050-5 33542245 PMC7862218

[40] Aldabbous MS , Roca MG , Stout A , Huang I-C , Read ND , Free SJ . 2010. The ham-5, rcm-1 and rco-1 genes regulate hyphal fusion in Neurospora crassa. Microbiology 156:2621–2629. doi:10.1099/mic.0.040147-0 20522492 PMC3068686

[41] Yamashiro CT , Ebbole DJ , Lee BU , Brown RE , Bourland C , Madi L , Yanofsky C . 1996. Characterization of rco-1 of Neurospora crassa, a pleiotropic gene affecting growth and development that encodes a homolog of Tup1 of Saccharomyces cerevisiae. Mol Cell Biol 16:6218–6228. doi:10.1128/MCB.16.11.6218 8887652 PMC231625

[42] Olivares-Yañez C , Emerson J , Kettenbach A , Loros JJ , Dunlap JC , Larrondo LF . 2016. Modulation of circadian gene expression and metabolic compensation by the RCO-1 corepressor of Neurospora crassa. Genetics 204:163–176. doi:10.1534/genetics.116.191064 27449058 PMC5012383

[43] Muñoz-Guzmán F , Caballero V , Larrondo LF . 2021. A global search for novel transcription factors impacting the Neurospora crassa circadian clock. G3 (Bethesda) 11:jkab100. doi:10.1093/g3journal/jkab100 33792687 PMC8495738

[44] Kelliher CM , Stevenson E-L , Loros JJ , Dunlap JC . 2023. Nutritional compensation of the circadian clock is a conserved process influenced by gene expression regulation and mRNA stability. PLoS Biol 21:e3001961. doi:10.1371/journal.pbio.3001961 36603054 PMC9848017

[45] Gonçalves RD , Cupertino FB , Freitas FZ , Luchessi AD , Bertolini MC . 2011. A genome-wide screen for Neurospora crassa transcription factors regulating glycogen metabolism. Mol Cell Proteomics 10:M111. doi:10.1074/mcp.M111.007963 PMC3226398 21768394

[46] Jonkers W , Fischer MS , Do HP , Starr TL , Glass NL . 2016. Chemotropism and cell fusion in Neurospora crassa relies on the formation of distinct protein complexes by HAM-5 and a novel protein HAM-14. Genetics 203:319–334. doi:10.1534/genetics.115.185348 27029735 PMC4858782

[47] Maddi A , Dettman A , Fu C , Seiler S , Free SJ . 2012. WSC-1 and HAM-7 are MAK-1 MAP kinase pathway sensors required for cell wall integrity and hyphal fusion in Neurospora crassa. PLoS One 7:e42374. doi:10.1371/journal.pone.0042374 22879952 PMC3411791

[48] Baryshnikova A , Costanzo M , Myers CL , Andrews B , Boone C . 2013. Genetic interaction networks: toward an understanding of heritability. Annu Rev Genomics Hum Genet 14:111–133. doi:10.1146/annurev-genom-082509-141730 23808365

[49] Watters MK , Manzanilla V , Howell H , Mehreteab A , Rose E , Walters N , Seitz N , Nava J , Kekelik S , Knuth L , Scivinsky B . 2018. Cold shock as a screen for genes involved in cold acclimatization in Neurospora crassa. G3 (Bethesda) 8:1439–1454. doi:10.1534/g3.118.200112 29563189 PMC5940138

[50] Ruger-Herreros C , Gil-Sánchez M del M , Sancar G , Brunner M , Corrochano LM . 2014. Alteration of light-dependent gene regulation by the absence of the RCO-1/RCM-1 repressor complex in the fungus Neurospora crassa. PLoS One 9:e95069. doi:10.1371/journal.pone.0095069 24747913 PMC3991626

[51] Olmedo M , Navarro-Sampedro L , Ruger-Herreros C , Kim S-R , Jeong B-K , Lee B-U , Corrochano LM . 2010. A role in the regulation of transcription by light for RCO-1 and RCM-1, the Neurospora homologs of the yeast Tup1–Ssn6 repressor. Fungal Genet Biol 47:939–952. doi:10.1016/j.fgb.2010.08.001 20709620

[52] Bastiaans E , Debets AJM , Aanen DK . 2016. Experimental evolution reveals that high relatedness protects multicellular cooperation from cheaters. Nat Commun 7:11435. doi:10.1038/ncomms11435 27139112 PMC4857390

[53] Westergaard M , Hirsch H . 1954. Environmental and genetic control of differentiation in Neurospora. Proc Symp Colson Res 7:171–183.

[54] Leslie JF , Summerell BA . 2006. The Fusarium laboratory manual. Blackwell Publishing, Hoboken, NJ. doi:10.1002/9780470278376

[55] Mylyk OM , Threlkeld SF . 1974. A genetic study of female sterility in Neurospora crassa. Genet Res 24:91–102. doi:10.1017/s001667230001510x 4279838

[56] McCluskey K , Wiest AE , Grigoriev IV , Lipzen A , Martin J , Schackwitz W , Baker SE . 2011. Rediscovery by whole genome sequencing: classical mutations and genome polymorphisms in Neurospora crassa. G3 (Bethesda) 1:303–316. doi:10.1534/g3.111.000307 22384341 PMC3276140

[57] Perkins DD . 1986. Hints and precautions for the care, feeding and breeding of Neurospora. Fungal Genet Rep 33:35. doi:10.4148/1941-4765.1588

